# Overall morbidity after total minimally invasive keyhole oesophagectomy versus hybrid oesophagectomy (the MICkey trial): study protocol for a multicentre randomized controlled trial

**DOI:** 10.1186/s13063-023-07134-1

**Published:** 2023-03-10

**Authors:** Rosa Klotz, Markus K. Diener, Thomas Schmidt, Thilo Hackert, Sandra Graf, Hans F. Fuchs, Peter Grimminger, Jan-Hendrick Egberts, Ines Gockel, Pieter C. van der Sluis, Colette Doerr-Harim, Christina Klose, Manuel Feißt, Andre L. Mihaljevic

**Affiliations:** 1 Department of General, Visceral and Transplantation Surgery, University Hospital Heidelberg, Im Neuenheimer Feld 420, 69120 Heidelberg, Germany; 2 Department of Surgery, Medical Center University Hospital Freiburg, Hugstetter Straße 55, 79106 Freiburg, Germany; 3 Department of General, Visceral, Tumor and Transplantation Surgery, University Hospital Cologne, Kerpener Str. 65, 50923 Cologne, Germany; 4 Department of General and Visceral Surgery and Clinical Trial Centre Department of Surgery (ulmCARES), University Hospital Ulm, Albert-Einstein-Allee 23, 89081 Ulm, Germany; 5 Department of General, Visceral and Transplantation Surgery, Universitätsmedizin der Johannes Gutenberg-Universität Mainz, Langenbeckstraße 1, 55131 Mainz, Germany; 6 Department of Surgery, Israelitisches Krankenhaus Hamburg, Orchideenstieg 14, 22297 Hamburg, Germany; 7 Clinic and Polyclinic for Visceral, Transplant, Thoracic and Vascular Surgery, University Hospital Leipzig, Liebigstraße 20, 04103 Leipzig, Germany; 8 Erasmus Medical Centre, Dr. Molewaterplein 40, 3015 GD Rotterdam, The Netherlands; 9 Institute of Medical Biometry (IMBI), University of Heidelberg, Im Neuenheimer Feld 130.3, 69120 Heidelberg, Germany

**Keywords:** Oesophageal neoplasms, Cancer of the oesophagogastric junction, Oesophagectomy, Patient-reported outcome measures, Postoperative complications, Randomized controlled trial

## Abstract

**Background:**

Oesophageal cancer (EC) is the sixth leading cause of cancer death worldwide. Oesophageal resection is the only curative treatment option for EC which is frequently performed via an abdominal and right thoracic approach (Ivor-Lewis operation). This 2-cavity operation is associated with a high risk of major complications. To reduce postoperative morbidity, several minimally invasive techniques have been developed that can be broadly classified into either hybrid oesophagectomy (HYBRID-E) via laparoscopic/robotic abdominal and open thoracic surgery or total minimally invasive oesophagectomy (MIN-E). Both, HYBIRD-E and MIN-E, compare favourable to open oesophagectomy. However, there is still an evidence gap comparing HYBRID-E with MIN-E with regard to postoperative morbidity.

**Methods:**

The MICkey trial is a multicentre randomized controlled superiority trial with two parallel study groups. A total of 152 patients with oesophageal cancer scheduled for elective oesophagectomy will be randomly assigned 1:1 to the control group (HYBRID-E) or to the intervention group (MIN-E). The primary endpoint will be overall postoperative morbidity assessed via the comprehensive complication index (CCI) within 30 days after surgery. Specific perioperative parameters, as well as patient-reported and oncological outcomes, will be analysed as secondary outcomes.

**Discussion:**

The MICkey trial will address the yet unanswered question whether the total minimally invasive oesophagectomy (MIN-E) is superior to the HYBRID-E procedure regarding overall postoperative morbidity.

**Trial registration:**

DRKS00027927 U1111-1277-0214. Registered on 4th July 2022

**Supplementary Information:**

The online version contains supplementary material available at 10.1186/s13063-023-07134-1.

## Administrative information

Note: the numbers in curly brackets in this protocol refer to SPIRIT checklist item numbers. The order of the items has been modified to group similar items (see http://www.equator-network.org/reporting-guidelines/spirit-2013-statement-defining-standard-protocol-items-for-clinical-trials/).

**Table:** 

Title {1}	Overall morbidity after total minimally invasive keyhole esophagectomy versus hybrid esophagectomy (the MICkey trial): study protocol for a multicentre randomized controlled trial
Trial registration {2a and 2b}.	DRKS00027927
Protocol version {3}	Version 1.0 8^th^ April 2022
Funding {4}	German Federal Ministry of Education and Research (Bundesministerium für Bildung und Forschung, BMBF). Funding number 01KG2028
Author details {5a}	Sandra Graf, Colette Doerr-Harim, Andre L. Mihaljevic: Department of General and Visceral Surgery and Clinical Trial Centre Department of Surgery (ulmCARES), University Hospital Ulm, Albert-Einstein-Allee 23, 89081 Ulm, Germany Thilo Hackert, Rosa Klotz: Department of General, Visceral and Transplantation Surgery, University Hospital Heidelberg, Im Neuenheimer Feld 420, 69120 Heidelberg, Germany Markus K. Diener: Department of Surgery, Medical Center University Hospital Freiburg, Hugstetter Straße 55, 79106 Freiburg, Germany Thomas Schmidt, Hans F. Fuchs: Department of General, Visceral, Tumor and Transplantation Surgery, University Hospital Cologne, Kerpener Str. 65, 50923 Cologne, Germany Peter Grimminger: Department of General, Visceral and Transplantation Surgery, Universitätsmedizin der Johannes Gutenberg-Universität Mainz, Langenbeckstraße 1, 55131 Mainz, Germany Jan-Hendrick Egberts: Israelitisches Krankenhaus Hamburg, Department of Surgery, Orchideenstieg 14, 22297 Hamburg, Germany Ines Gockel: Clinic and Polyclinic for Visceral, Transplant, Thoracic and Vascular Surgery, University Hospital Leipzig, Liebigstraße 20, 04103 Leipzig, Germany Pieter C. van der Sluis, Erasmus Medical Centre, Dr. Molewaterplein 40, 3015 GD Rotterdam, The Netherlands Manuel Feißt, Christina Klose: Institute of Medical Biometry (IMBI), University of Heidelberg, Im Neuenheimer Feld 130.3, 69120 Heidelberg
Name and contact information for the trial sponsor {5b}	Andre Mihaljevic: Department of General and Visceral Surgery, Clinical Trial Centre Department of Surgery (ulmCARES), University Hospital Ulm, Albert-Einstein-Allee 23, 89081 Ulm, Germany Phone: +49 (0) 0731-500-53502 Fax: +49-(0) 731 500-53503 E-mail: andre.mihaljevic@uniklinik-ulm.de
Role of sponsor {5c}	The funding body (German Ministry of Research and Education, Bundesministerium für Bildung und Forschung BMBF, Funding number 01KG2028) has NO role in study design; collection, management, analysis, and interpretation of data; writing of the report; and the decision to submit the report for publication. Furthermore, it has no ultimate authority over any of these activities.

## Introduction

### Background and rationale {6a}

Oesophageal cancer (EC) is the sixth leading cause of cancer death and the eleventh most common cancer worldwide [1]. Overall survival among patients with EC remains poor with an overall survival of 18%. The only curative treatment of EC is oesophageal resection, frequently in combination with other multimodal treatments. For resectable patients, the 3-year survival is around 50% [2, 3]. In Germany, 3500 resections of the oesophagus are performed each year [4]. Significant improvements in surgical practice and centralization in high-volume centres have reduced perioperative mortality after oesophagectomy to 5%. Yet, the risk for a major complication is as high as 50% [5–8] which leads to prolonged postoperative recovery, reduced health-related quality of life and increased costs [9]. Therefore, interventions that improve overall postoperative morbidity are urgently needed.

Due to the anatomical position of the oesophagus, a two-cavity oesophagectomy via abdominal and right thoracic approach (Ivor-Lewis procedure) and thoracic and abdominal lymphadenectomy is required for tumour resection. This can be either done via open surgery (with laparotomy and right thoracotomy) or via total minimally invasive oesophagectomy (MIN-E; either via “classical” minimally invasive laparoscopy and thoracoscopy; or via robotic-assisted minimally invasive oesophagectomy [RAMIE] or any combination of the two). Alternatively, a hybrid procedure (HYBRID-E) via laparoscopic/robotic abdominal surgery and open thoracic surgery is possible. Until recently, open oesophagectomy represented the standard procedure. However, over the last years, results from a number of randomized controlled trials (RCTs) have challenged this notion and have established MIN-E or HYBRID-E as new standard procedures for Ivor-Lewis oesophagectomy due to its reduced complication rates compared to open oesophagectomy.

Due to the lack of high-quality RCTs directly comparing HYBRID-E and MIN-E, it is unclear whether MIN-E represents an additional advantage over HYBRID-E in terms of postoperative morbidity. The available literature on MIN-E compared to HYBRID-E is heterogeneous with different surgical techniques and a high risk of bias due to the retrospective character of the analyses, small numbers of patients and historical cohorts [5, 7, 8, 10, 11]. On the one hand, a retrospective case series comparing MIN-E to a historic control group consisting of a patient population of open and HYBRID oesophagectomy revealed superiority for MIN-E regarding overall survival, perioperative mortality and severity of postoperative complications. This study exhibits a high risk of bias [12]. Another large retrospective comparative study with three groups (open versus MIN-E versus HYBRID-E) revealed improved survival in the MIN-E but not in the HYBRID-E group compared to the open procedure, again with a high risk of bias due to lack of adjustment for confounder variables [13]. A propensity score-matched comparison showed that MIN-E for EC reduces postoperative pain and pneumonia compared to HYBRID-E, but showed no difference in terms of postoperative surgical complications [14]. On the other hand, in a prospective comparative study including 315 patients, published as a congress abstract, a trend towards higher rates of overall complications and anastomotic leaks (without statistical significance) was reported for MIN-E compared to HYBRID-E [15]. In summary, data from a high-quality RCT comparing MIN-E versus HYBRID-E are lacking.

Therefore, according to the IDEAL framework, a multicentre RCT is indicated to test the comparative effectiveness of MIN-E vs. HYBRID-E (IDEAL stage 3; assessment) [16]. The MICkey trial will compare HYBRID-E vs. MINE-E in patients undergoing elective Ivor-Lewis oesophagectomy in respect to overall postoperative morbidity.

## Objectives {7}

The objective of the MICkey trial is to evaluate whether total minimally invasive Ivor-Lewis procedure (MIN-E, total minimally invasive abdominal-thoracic oesophagectomy) is superior to hybrid Ivor-Lewis procedure (HYBRID-E, minimally invasive abdominal surgery and open thoracic surgery) regarding overall postoperative morbidity measured via the comprehensive complication index [17, 18].

## Trial design {8}

MICkey is a multicentre, randomized controlled, patient-blinded superiority trial with two parallel study groups.

## Methods: participants, interventions and outcomes

### Study setting {9}

The trial will be performed by the Clinical Trials Network of the German Surgical Society (CHIR-*Net*, www.chir-net.de). To enrol the required number of patients in the planned recruitment period, eight trial sites with high expertise will participate. A list of study sites can be found in Supplement 1. Centres were chosen based on their expertise in oesophageal surgery. All centres must perform more than 26 oesophagectomies per year. Participating surgeons must have a lifetime experience of 40 MIN-E and/or 40 HYBRID-E.

### Eligibility criteria {10}

#### Inclusion and exclusion criteria for patients

All patients with a malignant tumour of the oesophagus or the oesophagogastric junction considered to be resectable with curative intent via oesophagectomy by means of an abdominal and right thoracic approach (Ivor-Lewis procedure) irrespective of neoadjuvant therapy are eligible for the study. All subjects must be suitable for the MIN-E and for the HYBRID-E procedure. Only adult patients (≥18 years of age) with the ability to understand character and individual consequences of the clinical trial will be included. All subjects must provide a written informed consent.

The preoperative exclusion criteria are defined as the presence of distant metastases, tumour localization above the azygos vein, history of right thoracotomy within the last 3 years, American Society of Anesthesiologists (ASA) grade >3 and advanced hepatic cirrhosis (Child B/C). Patients who participate in another intervention trial with interference of the intervention and/or primary outcome of the MICkey trial will be excluded as well as patients with an expected lack of compliance or language problems. Furthermore, two intraoperative exclusion criteria are defined: (a) intraoperative diagnosis of previously occult metastases that prohibit surgical resection according to current S3 guidelines [19] and (b) the tumour resection is technically impossible. For further handling of these cases (i.e., intraoperative exclusion of previously randomized patients), please refer to the section “Criteria for discontinuing or modifying allocated interventions {11b}”.

#### Eligibility criteria for trial sites and surgeons

The eligibility criterion for each participating trial site is the commitment to include ≥ 10 cases per year and can thus be regarded as major oesophageal cancer surgery centres [20]. This criterion is irrespective of the actual recruitment. Furthermore, all surgeons must have performed a minimum of 40 MIN-E or 40 HYBRID-E respectively to participate in the trial.

### Who will take informed consent? {26a}

All patients scheduled for an abdomino-thoracic oesophagectomy will be informed by an investigator, orally and written, of the aims of the study, the possible risks, the procedures, and possible hazards to which he/she will be exposed, and the mechanism of treatment allocation (randomization). The written informed consent form will be signed and personally dated by the patient according to the ICH guidelines on Good Clinical Practice.

### Additional consent provisions for collection and use of participant data and biological specimens {26b}

No biological samples will be collected during the trial.

## Interventions

### Explanation for the choice of comparators {6b}

Patients in both groups will receive oesophagectomy for their underlying tumour disease either via MIN-E or HYBRID-E. As pointed out in the “Introduction” section, both procedures have been shown to result in better outcomes than open oesophagectomy and can thus be regarded current standard of care. No high-level evidence directly compares the MIN-E and HYBRID-E directly. Therefore, a real clinical equipoise exists between the two comparators (see the “Introduction” section).

### Intervention description {11a}

#### Experimental and control intervention

In the experimental group, both the abdominal and thoracic phases of the elective oesophagectomy will be performed minimally invasively, i.e. as total minimally invasive oesophagectomy (MIN-E). MIN-E can be performed either via “classical” minimally invasive laparoscopy and thoracoscopy or via robotic-assisted minimally invasive oesophagectomy (RAMIE) or any combination of the two. A recent international consensus study has developed an operation manual for oesophagectomy [21]. The following steps are an adaptation of this consensus and serve as an operation recommendation for both the experimental and control interventions. Surgeons in the MICkey trial may deviate from this manual and should perform the procedure according to their local standard, i.e. according to the technique in which the operating surgeon has reached the plateau of her/his learning curve. In the following, the lymph node sections are named according to the Japan Esophageal Society and Japanese Gastric Cancer Association.

#### Abdominal phase

##### Step 1: Abdominal access

After a safe access to the abdominal cavity, confirmation of the absence of metastatic disease and judgement of the technical resectability is mandatory.

##### Step 2: Hepatoduodenal ligament and celiac axis 

Dissection of lymph node tissue along the common hepatic artery (LN 8a and optional 8p), the celiac artery (LN 9), the left gastric artery (LN 7) and the proximal splenic artery (LN 11p) is mandatory. The dissection of lymph node tissue along the proper hepatic artery (LN 12a) and the left side of the portal vein (LN 12p) is optional. The left gastric vein (close to the portal vein) and the left gastric artery (at its origin from the celiac artery) must be ligated and divided, followed by the dissection of the lymph node tissue from the left side of the celiac artery to the left crus at the oesophageal hiatus and left side of Gerota’s fascia.

##### Step 3: Gastric mobilization

Division of the greater omentum to enter the lesser sac, ensuring that the right gastroepiploic vessels are preserved to provide the blood supply to the gastric tube. Dissection along the greater curvature of the stomach towards the spleen, dividing the short gastric and left gastroepiploic vessels until the left diaphragmatic crus is reached. In doing so, the associated lymph node tissue (LN stations 4sa and 4sb) remains attached to the later specimen.

##### Step 4: Splenic artery

Ligation of the posterior gastric vessels if present at their origin. Afterwards, the optional dissection of the lymph node tissue along the anterior surface of the splenic artery (LN 11d) and the splenic hilum (LN 10) can be performed.

##### Step 5: Gastric tube formation

The lesser curvature of the stomach is cleared of LN tissue at the appropriate level, until the expected distal resection margin is reached (LN stations 3a and 3b). The gastric tube is created according to local standards (usually with linear staplers). This may also be performed in the chest depending on the local standard. The staple line of the gastric conduit can be oversewn if standard in the centre. A pyloroplasty, pyloromyotomy, or other may be performed if necessary.

##### Step 6: Diaphragmatic hiatus

Mobilization of the oesophagus from the diaphragmatic hiatus (LN 20) to the gastroesophageal junction (GEJ), resecting the right and left paracardial lymphatic (LN) tissue (LN 1 and 2). Transhiatal mobilization of the distal thoracic oesophagus with its surrounding (LN 110). Dissect along the pericardial adventitia to remove the pericardial tissue (LN 111). In advanced diseases, the right and left pleura can be resected. Dissect along the pre-aortic fascia (LN16a1 and 112aoA).

##### Step 7: Surgical adjuncts and closure 

Mandatory are the confirmation of haemostasis and the closure of the abdomen. Optional are the placement of a feeding jejunostomy, abdominal drain(s) and an abdominal lavage.

#### Thoracic phase

##### Step I: Thoracic access

The thoracic phase starts with a safe access to the patient’s right chest according to local standards.

##### Step II: Thoracic lymphadenectomy

Ligation and division of the azygos arch and divide the inferior pulmonary ligament. The dissection along the pericardium until the left lung is reached, including resection of the left pleura in advanced disease to achieve a clear circumferential margin is mandatory. Dissection is continued until the transhiatal dissection plane (abdominal part) is reached. Identify and ligate the thoracic duct above the level of the diaphragm, such that it is resected with the specimen. Perform a sub-carinal lymphadenectomy (LN 107). Clear both bronchi of LN tissue (LN 109). The dissection along the right pulmonary veins, continuing posteriorly until the left pulmonary veins are reached, is optional. Dissection of the mediastinal pleura at the anterolateral border of the thoracic aorta and the pre-aortic fascia, from the proximal resection margin towards the diaphragm (LN station 112). Dissection of lymph node tissue along the aorto-pulmonary window, clearing the arch of the aorta, pulmonary artery and recurrent laryngeal nerve as it hooks around the arch of the aorta (106tbL, 106recL, 105), is optional. Ligation of the thoracic duct at the proximal resection margin.

##### Step III: Specimen excision

Ensure that the thoracic part of the specimen is circumferentially free, from the previously completed diaphragmatic mobilization (performed during the abdominal phase) to above the level of the azygos vein (LN 108, 110 and 111). Deliver the stomach into the right chest cavity, ensuring that the gastric tube can reach the site of anastomosis without tension or torsion. Excise the specimen with suitable proximal and distal resection margins via the preferred way.

##### Step IV: Thoracic anastomosis

Perform an oesophagogastrostomy using your preferred method (stapler or suture).

##### Step V: Surgical adjuncts and closure

A nasogastric or nasojejunal tube may be placed. Thoracic drain(s) may be placed prior to the closure of the thoracic incision. After confirmation of haemostasis, the lung must be re-inflated under direct vision. Closure of the chest should be performed according to the local standard.

In the control group, the elective oesophagectomy will be performed as HYBRID-E, i.e. the abdominal phase of the operation will be performed minimally invasively while the thoracic part will be performed as open surgery (via a thoracotomy). The essential surgical steps performed in the control intervention are the same as for the experimental intervention and are outlined above.

### Criteria for discontinuing or modifying allocated interventions {11b}

Conversion to open surgery cannot be completely avoided, since reasons for conversion may arise intraoperatively, e.g. technical difficulties or bleeding. A low number of conversions reflect clinical reality and will not disappear even after the completion of the learning curve. Reasons for conversion will be captured for further evaluation of this subgroup. An overall conversion rate of <5% is expected for each study group. Patients undergoing conversion to open surgery (abdominal or thoracic part) remain in the trial and will be analysed in the modified intention-to-treat population.

If another type of surgery (other than abdominal and right thoracic oesophagectomy, Ivor-Lewis procedure) is indicated for complete tumour resection (e.g. transhiatal extended gastrectomy or abdomino-cervical oesophagectomy), the patient remains in the trial and will be followed up for the entire trial period. This is meaningful, because these types of surgeries constitute major operations, are associated with major complications and oncological outcome parameters can be analysed. The MICkey trial randomization will occur preoperatively, as intraoperative randomization is impractical due to the need for different operative set-ups (e.g. preparation of robot/laparoscopic towers) and patient preparation. To minimize bias, two intraoperative exclusion criteria have been defined. As a consequence, intraoperative exclusion of previously randomized patients might occur:

- *Case 1 (intraoperative diagnosis of previously occult metastases that prohibit surgical resection according to current S3 guidelines)* [19]: In this case, patients in both groups will undergo diagnostic laparoscopy only, as the abdominal part of the operation is the same in both groups and will be performed minimally invasive in both groups. Diagnostic laparoscopy is a minor surgery, with very few associated complications. Consequently, follow-up for the primary endpoint (comprehensive complication index) makes no sense, as it adds nothing to answering the primary research question, namely which surgical technique is associated with less complications after oesophagectomy, an operation which is associated with major complications. In addition, almost all secondary endpoints are meaningless in patients undergoing diagnostic laparoscopy only. Therefore, these patients will be excluded from the study. These patients will be defined as “intraoperative drop-out”. However, in line with the current S3 guidelines [19], patients with newly diagnosed intraoperative “sehr limitierter Fernmetastasen” (very limited metastases), which are “gut resektabel” (easily resectable), oesophagectomy in combination with resection of this “kleine, gut resektable Metastase” (small, well-resectable metastasis) should be performed [9] (recommendation 8.13). In this case, the patient remains in the trial and is regularly followed up.
- *Case 2 (tumour resection technically impossible)*. As for case 1, follow-up of patients makes little sense as these patients will undergo minor surgery only and DFS cannot be measured. Again, these patients will be defined as “intraoperative drop-outs”.
- The occurrence of both cases (case 1 and case 2) is assumed to be equally distributed between both study groups.

### Strategies to improve adherence to interventions {11c}

As MICkey is investigating two surgical interventions, strategies to improve adherence to the intervention are unnecessary as the patient cannot “withdraw” from the intervention during surgery.

### Relevant concomitant care permitted or prohibited during the trial {11d}

Additional treatments including all perioperative procedures are performed according to institutional standards and will be recorded to adjust for possible confounders in both groups.

### Provisions for post-trial care {30}

The post-trial care of the participants will be analogous to the regular tumour follow-up. Compensation is not provided.

### Outcomes {12}

#### Primary outcome/endpoint and assessment of primary outcome

The primary endpoint is overall postoperative morbidity within 30 days postoperatively measured via the comprehensive complication index (CCI) [17, 18]. The CCI ranges from 0 to a maximum of 100; the index expresses the cumulative morbidity that the patient experiences. The CCI is based on the established Dindo-Clavien classification (DCC) [22, 23]. A complication is any deviation from the normal postoperative course. The score is validated for oesophageal surgery, and a difference of 10 is regarded as a clinically relevant difference [18]. An endpoint reflecting the entire spectrum of complications like the CCI is highly appropriate to compare two different strategies like in the MICkey trial. The score will be calculated comprising all complications within 30 days.

A list of oesophagectomy-specific complications will be provided for all trial centres to facilitate documentation and assessment of postoperative morbidity according to the Esophagectomy Complications Consensus Group (ECCG) and the Dindo-Clavien grading system (Supplement 2) [24].

As postoperative oesophagogastroduodenoscopies (EGD) are frequent after oesophagectomy and performed routinely in some centres, a purely diagnostic EGD showing no pathological finding does *not* constitute a complication in the MICkey trial. Only if an endoscopic intervention (e.g. EndoVAC placement) is performed or a pathologic finding is identified on EGD (e.g. anastomotic leakage defined as “a full thickness GI defect involving oesophagus, anastomosis, staple line, or conduit irrespective of presentation or method of identification”) does this constitute a complication in the MICkey trial.

In the Addendum to the ICH E9 guideline, the estimand framework is recommended as a clear and transparent definition of “what is to be estimated” (International Council for Harmonization, 2019). An estimand is defined through the treatment, the targeted population, the variable (i.e. the endpoint), a specification of how to handle intercurrent events (post-randomization events) and a population-level summary. In the following, the primary estimand corresponding to the primary objective is described.

#### Treatment

Total minimally invasive oesophagectomy (MIN-E, either via “classical” minimally invasive laparoscopy and thoracoscopy or via robotic-assisted minimally invasive oesophagectomy [RAMIE] or any combination of the two) (experimental arm) vs. HYBRID-E (laparoscopic/robotic abdominal surgery and open thoracic surgery) (control arm)

#### Population

The targeted population is defined through the inclusion and exclusion criteria (incl. intraoperative exclusion criteria).

#### Variable

Overall postoperative morbidity in terms of the comprehensive complication index (CCI) within 30 days after surgery

#### Intercurrent events (ICE)

Specific events (e.g. death, re-operation) which can occur after randomization will be handled within the primary endpoint definition reflecting a composite strategy. Intraoperative deaths will be graded as grade V complication according to Dindo-Clavien. Treatment switcher from laparoscopic/robotic to open can occur and will be handled according to the intention-to-treat principle (treatment policy approach). Other intraoperative events can lead to an exclusion from the population (see intraoperative exclusion criteria).

Other post-randomization events will not be considered. This reflects a treatment policy approach, which means estimating the effect of randomized treatment irrespectively of other post-randomization events not captured in the primary endpoint definition.

#### Summary measure

The difference in mean CCI between study groups

#### Secondary outcomes/endpoints and assessment of secondary outcomes

1. 90-day mortality
2. Rate of re-operations within 90 days related to the index operation. Not included are planned elective surgeries not related to the index operation, e.g. port implantation.
3. Pulmonary complications within 30 days postoperative according to Esophagectomy Complications Consensus Group (ECCG) definition [24]. See Supplement 2 for definitions and grading of pulmonary complications.
4. Anastomotic leak (AL) defined according to ECCG as “full thickness GI defect involving oesophagus, anastomosis, staple line, or conduit irrespective of presentation or method of identification” within 30 days [24]. See Supplement 2 for grading of AL according to ECCG and DCC.
5. Conduit necrosis/failure within 30 days postoperative according to ECCG definition [24]. See Supplement 2 for grading of conduit necrosis/failure.
6. Delayed conduit emptying requiring intervention or delaying discharge or requiring maintenance of nasogastric drainage > 7 days (ECCG definition) within 30 days postoperative [24].
7. Chyle leak according to ECCG definition within 30 days [24]. See Supplement 2 for grading of the conduit chyle leak.
8. Recurrent nerve injury (vocal cord palsy) according to ECCG definition within 30 days [24]. See Supplement 2 for grading of recurrent nerve injury.

#### Surgical outcome measures

9. Operative time (in minutes; from the start of first skin incision until the closure of last skin incision). The time for repositioning of the patient between abdominal and thoracic part is included.
10. Length of hospital stay (in days from the day of surgery until discharge)
11. Conversion to open surgery. Conversion to open surgery during any of the minimally invasive parts of the operation (abdominal or thoracic). Reasons for conversion will be documented in the eCRF.

#### Patient-reported outcome measures (PROM)

12. Overall pain at rest and during movement (Numeric Rating Scale, NRS) at visits 3–5. The NRS ranges from 0 “no pain” to 10 “pain as bad as you can imagine” or “worst pain imaginable”.
13. Assessment of postoperative thoracic pain syndrome measured as pain in the chest at rest and while coughing (NRS, 0–10) at visits 3–10. Chronic postoperative thoracic pain syndrome is a common and debilitating complication after thoracic surgery and was thus included as an endpoint in our trial. HYBRID-E and MIN-E might have different rates of thoracic pain syndrome due to differences in surgical access.
14. Quality of recovery according to QoR-15 at visits 3–5 [25]
15. Quality of life measured via QLQ-C30 and QLQ-OES18 at visits 7–10

#### Oncological outcome measures

16. Total lymph node count
17. Rate of microscopic negative resection margin (R0)
18. Disease-free survival (DFS) defined as the time from randomization until disease recurrence (local recurrence or metastases) or death from any cause within 2 years. DFS was chosen because it is clinically one of the most relevant oncologic outcome parameters according to regulatory agencies [26, 27].
19. Overall survival (OS) defined as the time between randomization and death from any cause within 2 years. OS was chosen because it is clinically one of the most relevant oncologic outcome parameters according to regulatory agencies [26, 27].

#### Safety measures

20. Rate of serious adverse events defined as postoperative complication Clavien-Dindo grades III–V within 90 days after surgical intervention.

### Participant timeline {13}

**Table:** 

**Visit**	**V1**	**V2**	**V3**–**5**	**V6**	**V7**	**V8**	**V9/V10**
	Before surgery	Surgery (day 0)	POD 2 (+1) POD 4 (+1) POD 7 (+2)	Discharge	POD 30 (±5)	POD 90 (±5)	12 months postop. 24 months postop.
	**Outpatient/inpatient**	**Inpatient**			**Outpatient**	**Outpatient or telephone/mail**	
Eligibility criteria	X						
Informed consent	X						
Baseline demographics and clinical data	X						
Randomization	X						
Surgical data		X					
Histopathology (lymph node count, R0-rate)		X					
Assessment of primary endpoint (CCI)			X	X	X		
Assessment of reoperations			X	X	X	X	X
Safety (SAE)			X	X	X	X	
Assessment of secondary, oesophagectomy-specific outcomes according to ECCG			X	X	X		
Length of hospital stay				X			
Pain at rest and during movement (NRS)			X				
Thoracic pain syndrome measured as pain in the chest at rest and while coughing (NRS)			X	X	X	X	X
Quality of recovery (QoR-15)	X		X				
Health-related quality of life (EORTC QLQ-C30 + OES-18)	X				X	X	X
Disease-free survival					X	X	X
Overall survival (including 90-day mortality)		X	X	X	X	X	X

### Sample size {14}

**Fig. 1:**
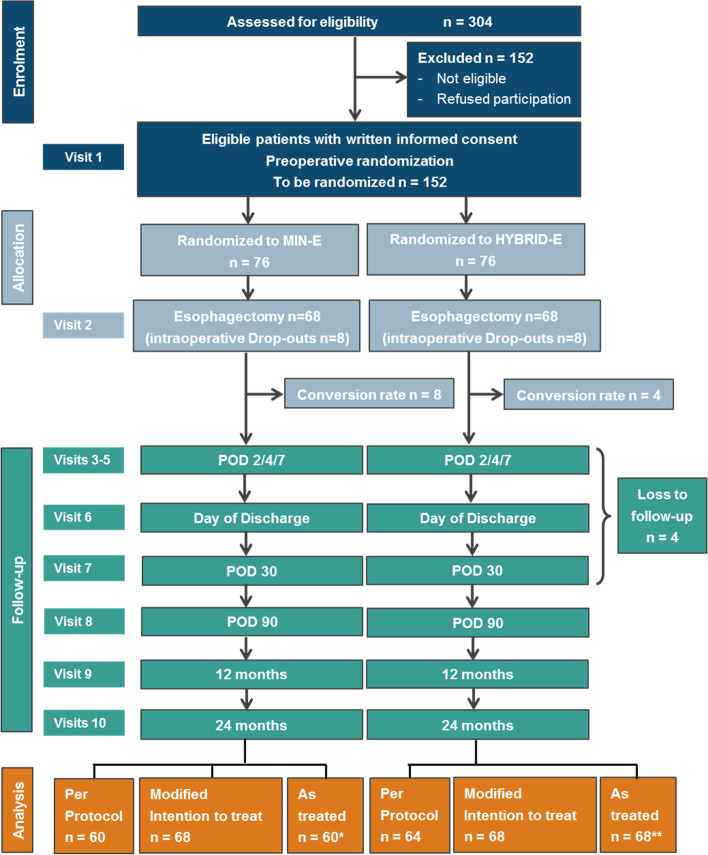
Flow chart. *MINE-E group: as treated excluding 8 converted. **HYBRID-E group: as treated including 4 converted from the experimental arm

The sample size calculation is based on the primary endpoint, CCI within 30 days after surgery. Assumptions are based on the literature: a decrease of the CCI by 10 points is considered relevant by patients and clinicians and a conservative standard deviation of 20 is assumed [1]. Based on a *t*-test with a two-sided significance level of *α*=0.05, a sample size of *n*=128 patients (64 per group) has to be recruited to achieve a power of 80% to reveal this difference. The primary endpoint will be analysed by a linear mixed regression model which leads to equal or even increased power as compared to using a two-sided *t*-test (see the “Statistical methods” section for further detail). To compensate for “intraoperative drop-outs” (10%; decision for no tumour resection) and “postoperative drop-out” and “loss to follow-up”, further 15% of patients will be randomized leading to a total sample size of *n*=152 (76 per group). The number of patients to be screened (*n*=304 to be assessed for eligibility; 304 × 0.5 = 152) was calculated with an assumed 50% of patients participating and meeting the inclusion and exclusion criteria (Fig. 1).

An intraoperative drop-out rate of 10% is assumed for the primary endpoint: only randomized patients who do not undergo oesophagectomy due to intraoperative findings will be counted as an intraoperative dropout. The amount of these patients is estimated to be 10% based on previous studies [5, 7]. A “postoperative drop-out” and “lost-to-follow-up” rate of 5% is assumed. Clinical visits after discharge will be done as outpatient or telephone visits if patients are unable to attend. Based on previous trials conducted by the Study Center of the German Society of Surgery and the CHIR-*Net*, the rate of “postoperative drop-out” and “lost-to-follow-up” is approximately 5% with a primary endpoint evaluated within 1 month [22].

### Recruitment {15}

To enrol the required number of patients in the planned recruitment period, 8 trial sites will participate in this trial. The trial will be performed by the Clinical Trial Network of the German Society of Surgery (CHIR-*Net*, www.chir-net.de). For a full list of participating centres, please see Supplement 1. Recruitment will start in March 2023 and run until February 2025. All patients with oesophageal cancer in the participating hospitals will be screened and asked to participate in the trial.

## Assignment of interventions: allocation

### Sequence generation {16a}

To ensure equal distribution of patient characteristics randomization will be used. Allocation of treatments will be performed using a web-based randomization tool (www.randomizer.at). Block-wise randomization of variable block sizes will be conducted. Randomization will be performed preoperatively for practicality reasons. Randomization will be stratified by centre.

### Concealment mechanism {16b}

Randomization will be performed preoperatively as planning, positioning and operative set-up differ between MIN-E and HYBRID-E. Block size will be kept confidential to the study team.

### Implementation {16c}

Randomization will be performed by a study team member before surgery. Names of the randomizing study team member as well as the operating surgeons will be noted. They will not be involved in outcome assessment.

## Assignment of interventions: blinding

### Who will be blinded {17a}

The following blinding measures will be implemented in the trial according to current recommendations [28]:

A. Patients will not be informed about the randomization result for the first 7 postoperative days (till after visit 5) to ensure blinding for the early postoperative period. They will be blinded with a large chest dressing until after visit 5 [3]. As the abdominal access is minimally invasive in both groups, no special blinding measures are needed for the abdomen. Accordingly, subjective outcomes prone to detection bias such as quality of recovery assessed on postoperative day (POD) 4 and POD 7 and pain at rest and during movement assessed on POD 2, 4 and 7 will be assessed while patients are still blinded. Long-term blinding of patients is not feasible, since unblinding during a change of wound dressings or during inspection of the wound is likely. However, most endpoints including the primary endpoint CCI are assessed according to objective criteria (e.g. reoperation, ICU stay, death) and are thus not affected by unblinding.
B. Surgeons performing the operation are unblinded to the trial intervention. Consequently, surgeons who have performed the operation should not act as outcome assessors to avoid influencing the primary endpoint.
C. Similarly, blinding of the treating team is not feasible, since unblinding during change of wound dressings would be likely. Again, endpoints other than the above-mentioned PROMs (pain, QoR) are not affected by unblinding as they are assessed according to objective criteria (e.g. reoperation, ICU stay, death) and are thus not affected by unblinding.
D. Blinding of outcome assessors is not necessary as endpoints other than the above-mentioned PROMs (pain, QoR) are not affected by unblinding as they are assessed according to objective criteria (e.g. reoperation, ICU stay, death) and are thus not affected by unblinding.

The trial statistician will perform the analyses according to a predefined statistical analysis plan which will be finished prior to database closure.

### Procedure for unblinding if needed {17b}

If unblinding of the patient is necessary in the first 7 postoperative days, this can be done by the unblinded study personnel or the treating physicians.

## Data collection and management

### Plans for assessment and collection of outcomes {18a}

See the section “Outcomes {12}”. The trial will be performed by the Clinical Trial Network of the German Society of Surgery (CHIR-*Net*, www.chir-net.de). The CHIR-*Net* has successfully performed trials with similar indications and recruitment rates. All trial sites have already successfully participated in surgical clinical trials and have the necessary expertise, equipment and personnel to perform this trial.

### Plans to promote participant retention and complete follow-up {18b}

In order to minimize loss to follow-up, the following measures have been implemented:

- A pragmatic trial design with high-external validity, meaning that trial sites will have little problems to adapt their standard clinical workflow to the follow-up visits
- The follow-up visits after discharge can be performed via telephone if patients are unable to attend an outpatient appointment
- All trial sites are trained in performing surgical clinical trials (CHIR-Net) and have successfully participated in similar trials
- Only high-volume trial sites committing to include at least 10 patients per year will be chosen as trial sites. The sites must perform 26 or more oesophagectomies annually.

### Data management {19}

All protocol-required information collected during the trial must be entered by the investigator, or designated representative, in the eCRF. The investigator, or designated representative, should complete the eCRF pages as soon as possible after information is collected, preferably on the same day that a trial subject is seen for an examination, treatment or any other trial procedure. Any outstanding entries must be completed immediately after the final examination. An explanation should be given for all missing data. Protocol deviations become clear via documentation in the eCRF and will be listed by the data management. Further protocol deviations are explicitly asked for in the eCRF and have to be described by trial centres. The completed eCRF must be reviewed and signed by the investigator named in the trial protocol or by a designated sub-investigator. The Heidelberg Institute of Medical Biometry (IMBI) is responsible for the data management within the trial. The study data will be collected and managed using REDCap (Research Electronic Data Capture) [29], a secure, web-based data capture application hosted at the IMBI. To assure a safe and secure environment for data acquired, data transmission is encrypted with secure socket layer (SSL) technology. Only authorized users are able to enter or edit data, and the access is restricted to data of the patients in the respective centre. All changes to data are logged with a computerized timestamp in an audit trail. All data will be pseudonymized. To guarantee high data quality, data validation rules will be defined in a data validation plan. Completeness, validity and plausibility of data will be checked in time of data entry (edit-checks) and using validating programs, which will generate queries. If no further corrections are to be made in the database, eCRF data will be locked. Data will finally be downloaded and used for statistical analysis. All data management procedures will be conducted according to written defined standard operating procedures (SOPs) of the IMBI that guarantee an efficient conduct complying with GCP. At the end of the study, the data will be transformed into different data formats (e.g. csv-files) for archiving and to ensure that it can be re-used.

### Confidentiality {27}

All information on study participants will be retained in password-protected files and locked cabinets at the Clinical Trials Centre. To ensure confidentiality, participants will be allocated an individual trial identification number. Access to this information will only be provided to immediate study staff, unless required by legislative or regulatory agencies.

### Plans for collection, laboratory evaluation and storage of biological specimens for genetic or molecular analysis in this trial/future use {33}

No biological samples are collected.

## Statistical methods

### Statistical methods for primary and secondary outcomes {20a}

For the examination of the primary endpoint, CCI within 30 days after surgery, the hypotheses to be assessed in the primary analysis are as follows:

H0: μ1 = μ2 vs H1: μ1 ≠ μ2, where μ1 and μ2 denote the mean CCI in the control and intervention groups respectively.

The confirmatory analysis of the primary efficacy endpoint corresponds to the primary estimand. Only patients who underwent oesophagectomy will be included in the primary analysis (drop-out assumed to be less than 10%) and they will be analysed in the group they were randomized (converted patients remain in their group). This reflects an analysis according to a modified intention-to-treat principle and the derived dataset is referred to as the mITT. The level of significance is set to 5% (two-sided).

The mean differences of the CCI will be examined using a linear mixed model including treatment group and adjusting for age as a fixed effect, as well as center as a random effect. Due to the stratified randomization and relatively large number of centers in relation to the sample size, inclusion of centre as a random effect is recommended [24]. The correlation matrix will have a variance components structure. The primary analysis will be performed after data for the primary endpoint is available for all patients. The database will be closed before the analysis of the primary endpoint.

### Interim analyses {21b}

No intermediate analyses are carried out.

### Methods for additional analyses (e.g. subgroup analyses) {20b}

In the following, supplementary analyses for the primary estimand are described: The same test as in the primary analysis will be conducted in the per-protocol set (PP, based on those patients without major protocol violation) and the as-treated set (AS, considers those subjects who have actually received the intervention, analysed in the group they were treated). Failure to respect the exclusion criteria or to follow the technical aspects of the assigned study group (suture material, stitch technique, used mesh) will be considered as major protocol violations. Analyses in the PP and AS set are known to be biased and do not correspond to any estimand and thus have to be interpreted with great caution. In addition, the primary endpoint will be compared between the study groups by the Mann-Whitney *U* test. In contrast to the primary endpoint model, the Mann-Whitney *U* test does not consider the mean CCI, but the “distribution of the values per se” and therefore does not address the primary estimand.

Pre-specified subgroup analysis will be performed in the following subgroups based on the mITT:

- Patients who switched from MIN-E to HYBRID-E (y/n)
- Patients with a tumour of the thoracic oesophagus versus a tumour of the oesophagogastric junction
- Squamous cell versus adenocarcinoma
- Robot-assisted procedures versus conventional minimally invasive procedures
- Adherence to ERAS items (yes/no)
- Neoadjuvant therapy (y/n)
- Further subgroups will be determined in the statistical analysis plan

In general, for the mITT, all baseline values will be evaluated descriptively per group and for the whole cohort. Furthermore, secondary endpoints will be evaluated descriptively and effect sizes will be reported together with 95% confidence intervals for the corresponding effects. In addition, regression models including the treatment group as the fixed effect and centre as random effect as specified for the primary endpoint will be used. Time-to-event endpoints will be evaluated by methods of survival analysis comprising the Kaplan-Meier method and Cox proportional hazards models. Secondary endpoint analysis will be performed on the mITT and the AS and no missing values will be imputed.

### Methods in analysis to handle protocol non-adherence and any statistical methods to handle missing data {20c}

In the primary analysis, missing data are assumed to be at least “missing at random” and will be replaced using multiple imputation using the fully conditional specification method [16] and predictive matching taking the variables treatment group, age, comorbidity index, ASA status, neoadjuvant treatment, tumour type and tumour stage.

Due to the nature of the primary endpoint and the short evaluation period, missing data in the primary endpoint model is assumed to be very rare. Therefore, no sensitivity analyses regarding the imputation method and the underlying assumptions will be performed.

### Plans to give access to the full protocol, participant-level data and statistical code {31c}

The full protocol is accessible with this publication. Participant-level data will be available anonymized after publication of the final results of the study.

## Oversight and monitoring

### Composition of the coordinating centre and trial steering committee {5d}

The steering committee will supervise the conduct of the trial and will issue recommendations for early termination, modifications or continuation of the trial, if necessary. The steering committee consists of the trial statistician as well as of clinical experts.

### Composition of the data monitoring committee, its role and reporting structure {21a}

An independent data and safety monitoring board (DSMB) will be set up to ensure the ethical conduct of the trial and protect the rights and welfare of the patients. Therefore, one of its 3 members is an experienced surgeon who is not part of the trial. The second member is an independent statistical expert and the third member is a patient representative. The DSMB will monitor and supervise the trial’s progress and will communicate on the state of the trial on a regular basis.

Members of the DSMB should advise whether to continue, modify or stop the trial based on the rate of complications. The DSMB will make recommendations to the steering committee on further conduct of the study, e.g. modification, continuation and closure. The data necessary for the DSMB to fulfil its function are provided by the data management on a regular basis.

The DSMB will supervise all safety data. Major postoperative complications (Clavien-Dindo grades III–V corresponding to serious adverse events) within 90 days, intraoperative deaths and intraoperative conversions from minimally invasive to open surgery notified by investigators will be compiled for and reviewed by the DSMB after the first 10 patients have undergone each procedure and visit 7 was performed for each of them. Depending on the results of this first and early safety evaluation, further safety analyses will be scheduled. The DSMB members will propose modifications, e.g. in study procedures, or precautions, if indicated. For the option of termination for the potential health hazard caused by the study treatment. Thus, the ongoing risk/benefit assessment of the trial will be ensured.

### Adverse event reporting and harms {22}

During the MICkey trial, adverse events are assessed as minor complications (Clavien-Dindo grade <3) within 30 days postoperative (primary endpoint).

Serious adverse events in the MICkey trial are defined and assessed as major postoperative complications Clavien-Dindo grades III–V within 90 days after the index operation. In addition, also intraoperative deaths and intraoperative conversions from minimally invasive to open surgery will be documented.

Since the MICkey trial is a clinical trial according to Medical Association’s professional code (Berufsordnung der Bundesärztekammer) § 15, no specific SAE management is required. However, a GCP-conform AE and SAE management is implemented. AE and SAEs will be collected via the primary and secondary endpoints directly in the EDC system.

### Frequency and plans for auditing trial conduct {23}

Periodic safety reports will be compiled during the respective time period of 90 days after the initial operation. The frequency of subsequent reports will depend on the results of the first DSMB evaluation.

### Plans for communicating important protocol amendments to relevant parties (e.g. trial participants, ethical committees) {25}

The ethics committee will be informed of all subsequent protocol amendments to determine whether formal approval must be sought and whether the informed consent document should also be revised.

## Dissemination plans {31a}

Trial results will be reported according to the CONSORT statement and the FAIR data principles. Publication in international open-access peer-reviewed journals is intended. Trial results will be communicated to participating trial sites prior to publication. Trial results will be presented at an international conference.

## Discussion

The incidence of EC is increasing in the Western world [30]. Survival among patients with EC remains poor with an overall survival of 18%. Even for patients who undergo curative surgery, the 3-year survival is around 50% [2, 3]. EC caused 9.3 million disability-adjusted life years (DALYs) in 2016 out of the overall 208.3 million DALYs caused by cancer worldwide [1]. It causes the seventh most absolute years of life lost (YLLs) of all cancers and 5% of all cancer-related deaths [1].

The MICkey trial is a multicentre randomized controlled trial assessing whether total minimally invasive Ivor-Lewis procedure (MIN-E) is superior to the hybrid Ivor-Lewis procedure (HYBRID-E) regarding overall postoperative morbidity.

There are already six RCTs with 822 patients comparing different types of minimally invasive to open oesophagectomy with a thoracic anastomosis for EC [9, 11, 12, 15–17]. Results of these trials have been synthesized in a recent meta-analysis [18]. In all 6 studies, minimally invasive techniques were compared to open surgery. In the single-centre study by van der Sluis et al., a robot-assisted totally minimally invasive approach was compared to open surgery [12]. In the trials by Straatman et al. [11], Ma et al. [27] and Guo et al. [13] the minimally invasive group consisted of laparoscopy and thoracoscopy (MIN-E). The studies by Mariette et al. [10] and Paireder et al. [19] compared HYBRID-E with open oesophagectomy.

While van der Sluis et al. and Guo et al. used cervical anastomotic techniques, the TIME trial [10, 11] employed a mixture of cervical and intrathoracic anastomoses. All other trials used intrathoracic anastomotic techniques.

In these trials, MIN-E has shown advantages compared to the open strategy with regard to complications, health-related quality of life and postoperative functional recovery [11, 20, 27]. Furthermore, no differences in disease-free and overall 3-year survival have been reported between minimally invasive approaches compared to open surgery [18].

The trials conducted by Paireder et al. and Mariette et al. compared HYBRID-E vs. open oesophagectomy. While the single-centre RCT by Paireder et al. was terminated prematurely due to a high number of consecutive anastomotic leakages in both groups [15], the high-quality multicentre RCT by Mariette et al. found that HYBRID-E resulted in a lower incidence of intra- and postoperative major complications, specifically pulmonary complications, than open oesophagectomy [9]. In summary, compared to open surgery both HYBRID-E and MIN-E seem to be superior.

However, due to the lack of high-quality RCTs directly comparing HYBRID-E and MIN-E, it is unclear whether MIN-E represents an additional advantage over HYBRID-E. The available literature on MIN-E compared to HYBRID-E is heterogeneous with different surgical techniques and a high risk of bias due to the retrospective character of the analyses, small numbers of patients and historical cohorts [19–22]. On the other hand, in a prospective comparative study including 315 patients, published as a congress abstract, a trend towards higher rates of overall complications and anastomotic leaks (without statistical significance) was reported for MIN-E compared to HYBRID-E [23].

In summary, data from a high-quality RCT comparing MIN-E versus HYBRID-E are lacking. The implementation of MIN-E into clinical routine might offer an improvement for patients regarding optimal recovery with less complications and a shorter length of hospital stay. Postoperative complications have been shown to be associated with lower long-term health-related quality of life [31]. Besides, complications in surgery are the major cost drivers [9]. Thus, aiming at a reduction of complications is both patient-oriented and economically sensible.

However, there is an evidence gap whether MIN-E is superior to HYBRID-E regarding postoperative complications. Therefore, according to the IDEAL framework of surgical innovations, a multicentre RCT is indicated to test the comparative effectiveness of MIN-E vs. HYBRID-E (IDEAL stage 3; assessment) [26].

## Trial status

This manuscript was written according to the most current version of the study protocol (version 1.0, last updated on April 08, 2022). Recruitment of patients for the MICkey trial will start in November 2022. The clinical phase of the trial (last patient out) is expected to be completed by the end of 2026.

## Supplementary Information

**Additional file 1: Supplement 1.** A list of study sites.

**Additional file 2: Supplement 2.** Common postoperative complications after esophagectomy and their respective definitions adapted according to the Esophagectomy Complications Consensus Group (ECCG). Grading according to Dindo-Clavien with clarifications for specific complications by the Japan Clinical Oncology Group.

## Data Availability

It is planned to make anonymous trial data on which scientific publications are based and all anonymous primary data publicly available for re- and meta-analyses after completion of the trial an appropriate repository.

## Abbreviations

CCI: Comprehensive complication index
eCRF: Electronic case report form
DCC: Dindo-Clavien classification
DRKS: Deutsches Register klinische Studien (German Clinical Trials Register)
DSMB: Data Safety Monitoring Board
EC: Oesophageal cancer
ICH: International Conference on Harmonization of Technical Requirements for Registration of Pharmaceuticals for Human Use
IEC: Independent Ethics Committee
IMBI: Institute of Medical Biometry
NRS: Numeric Rating Scale
POD: Postoperative day
QLQ: Quality of Life Questionnaire
QoR-15: Quality of Recovery 15
RCT: Randomized controlled trial
SAE: Serious adverse event
SC: Steering Committee
SOP: Standard operating procedure
V: Visit
ZKS Ulm: Clinical Trials Centre Ulm (Zentrum für Klinische Studien Ulm)

## References

[1] Slankamenac K, Graf R, Barkun J, Puhan MA, Clavien P-A (2013). The comprehensive complication index: a novel continuous scale to measure surgical morbidity. Ann Surg.

[2] Reeve JC, Nicol K, Stiller K, McPherson KM, Denehy L (2008). Does physiotherapy reduce the incidence of postoperative complications in patients following pulmonary resection via thoracotomy? A protocol for a randomised controlled trial. J Cardiothorac Surg.

[3] Mariette C, Dahan L, Mornex F, Maillard E, Thomas P-A, Meunier B (2014). Surgery alone versus chemoradiotherapy followed by surgery for stage I and II esophageal cancer: final analysis of randomized controlled phase III trial FFCD 9901. J Clin Oncol.

[4] Stark PA, Myles PS, Burke JA (2013). Development and psychometric evaluation of a postoperative quality of recovery score: the QoR-15. J Am Soc Anesthesiol.

[5] Blazeby J, Conroy T, Hammerlid E, Fayers P, Sezer O, Koller M (2003). Clinical and psychometric validation of an EORTC questionnaire module, the EORTC QLQ-OES18, to assess quality of life in patients with oesophageal cancer. Eur J Cancer.

[6] Mamidanna R, Ni Z, Anderson O, Spiegelhalter SD, Bottle A, Aylin P (2016). Surgeon volume and cancer esophagectomy, gastrectomy, and pancreatectomy. Ann Surg.

[7] Chang AC (2018). Centralizing esophagectomy to improve outcomes and enhance clinical research: invited expert review. Ann Thorac Surg.

[8] Abdelsattar ZM, Habermann E, Borah BJ, Moriarty JP, Rojas RL, Blackmon SH (2020). Understanding failure to rescue after esophagectomy in the United States. Ann Thorac Surg.

[9] Markar SR, Karthikesalingam A, Thrumurthy S, Low DE (2012). Volume-outcome relationship in surgery for esophageal malignancy: systematic review and meta-analysis 2000-2011. J Gastrointest Surg.

[10] Mariette C, Markar SR, Dabakuyo-Yonli TS, Meunier B, Pezet D, Collet D (2019). Hybrid minimally invasive esophagectomy for esophageal cancer. N Engl J Med.

[11] Straatman J, Van Der Wielen N, Cuesta MA, Daams F, Garcia JR, Bonavina L (2017). Minimally invasive versus open esophageal resection: three-year follow-up of the previously reported randomized controlled trial the TIME Trial. Ann Surg.

[12] Tapias LF, Morse CR (2014). Minimally invasive Ivor Lewis esophagectomy: description of a learning curve. J Am Coll Surg.

[13] Guo W, Zou Y-B, Ma Z, Niu H-J, Jiang Y-G, Zhao Y-P (2013). One surgeon’s learning curve for video-assisted thoracoscopic esophagectomy for esophageal cancer with the patient in lateral position: how many cases are needed to reach competence?. Surg Endosc.

[14] Hernandez JM, Dimou F, Weber J, Almhanna K, Hoffe S, Shridhar R (2013). Defining the learning curve for robotic-assisted esophagogastrectomy. J Gastrointest Surg.

[15] van der Wilk BJ, Hagens ERC, Eyck BM, Gisbertz SS, van Hillegersberg R, Nafteux P, Schröder W, Nilsson M, Wijnhoven BPL, Lagarde SM, van Berge Henegouwen MI (2022). International Esodata Study Group Collaborators. Outcomes after totally minimally invasive versus hybrid and open Ivor Lewis oesophagectomy: results from the International Esodata Study Group. Br J Surg.

[16] Van Buuren S, Brand JP, Groothuis-Oudshoorn CG, Rubin DB (2006). Fully conditional specification in multivariate imputation. J Stat Comput Simul.

[17] Jiang R, Liu Y, Ward KC, Force SD, Pickens A, Sancheti MS (2018). Excess cost and predictive factors of esophagectomy complications in the SEER-Medicare database. Ann Thorac Surg.

[18] Schlottmann F, Strassle PD, Patti MG (2017). Transhiatal vs. transthoracic esophagectomy: a NSQIP analysis of postoperative outcomes and risk factors for morbidity. J Gastrointest Surg.

[19] Paireder M, Asari R, Kristo I, Rieder E, Zacherl J, Kabon B (2018). Morbidity in open versus minimally invasive hybrid esophagectomy (MIOMIE). Eur Surg.

[20] van der Sluis PC, van der Horst S, May AM, Schippers C, Brosens LA, Joore HC (2019). Robot-assisted minimally invasive thoracolaparoscopic esophagectomy versus open transthoracic esophagectomy for resectable esophageal cancer: a randomized controlled trial. Ann Surg.

[21] Hackert T, Probst P, Knebel P, Doerr-Harim C, Bruckner T, Klaiber U (2018). Pylorus resection does not reduce delayed gastric emptying after partial pancreatoduodenectomy: a blinded randomized controlled trial (PROPP Study, DRKS00004191). Ann Surg.

[22] Diener MK, Seiler CM, Rossion I, Kleeff J, Glanemann M, Butturini G (2011). Efficacy of stapler versus hand-sewn closure after distal pancreatectomy (DISPACT): a randomised, controlled multicentre trial. Lancet..

[23] S3-Leitlinie Ösophaguskarzinom - Version 3.1, 2022, AWMF-Registernummer: 021/023OL. Available at: https://www.leitlinienprogramm-onkologie.de/fileadmin/user_upload/Downloads/Leitlinien/Oesophaguskarzinom/Version_3/LL_%C3%96sophaguskarzinom_Langversion_3.1.pdf

[24] Kahan BC, Harhay MO (2015). Many multicenter trials had few events per center, requiring analysis via random-effects models or GEEs. J Clin Epidemiol.

[25] Fitzmaurice C, Allen C, Barber RM, Barregard L, Bhutta ZA, Brenner H (2017). Global, regional, and national cancer incidence, mortality, years of life lost, years lived with disability, and disability-adjusted life-years for 32 cancer groups, 1990 to 2015: a systematic analysis for the global burden of disease study. JAMA Oncol.

[26] Vonlanthen R, Slankamenac K, Breitenstein S, Puhan MA, Muller MK, Hahnloser D (2011). The impact of complications on costs of major surgical procedures: a cost analysis of 1200 patients. Ann Surg.

[27] Ma G, Cao H, Wei R, Qu X, Wang L, Zhu L (2018). Comparison of the short-term clinical outcome between open and minimally invasive esophagectomy by comprehensive complication index. J Cancer Res Ther.

[28] Siegel RL, Miller KD, Jemal A (2017). Cancer statistics, 2017. CA Cancer J Clin.

[29] Statistisches Bundesamt OuPdvPuPiK, ausführliche Darstellung. 2017.

[30] Mariette C, Piessen G, Triboulet J-P (2007). Therapeutic strategies in oesophageal carcinoma: role of surgery and other modalities. Lancet Oncol.

[31] Cuschieri A, Shimi S, Banting S (1992). Endoscopic oesophagectomy through a right thoracoscopic approach. J R Coll Surg Edinb.

