# Supplementary material for: Overall morbidity after total minimally invasive keyhole oesophagectomy versus hybrid oesophagectomy (the MICkey trial): study protocol for a multicentre randomized controlled trial
Source: Trials. 2023 Mar 10;24:175. doi: 10.1186/s13063-023-07134-1 (PMC9999550; doi:10.1186/s13063-023-07134-1)
Supplement: Supplementary file 1 — Additional file 1: Supplement 1. A list of study sites. [file 13063_2023_7134_MOESM1_ESM.docx]

| **Zentrum** | **Trial Site** | **PI** |
| --- | --- | --- |
| Universitätsklinikum Freiburg  Department Chirurgie  Klinik für Allgemein- und Viszeralchirurgie | Department of General and Visceral Surgery  Department of Surgery, Medical Center  University of Freiburg | Prof. Dr. med. Markus Diener |
| Israelitisches Krankenhaus Hamburg  Chirurgische Klinik  Viszeral-Medizinisches Zentrum | Israeli Hospital Hamburg  Department of Surgery  Visceral Medical Center | Dr. med. Margret Alm |
| Universitätsklinikum Heidelberg  Klinik für Allgemein-, Viszeral- und Transplantationschirurgie | University Hospital Heidelberg  Department of General, Visceral and Transplantation Surgery | PD Dr. med. Rosa Klotz |
| Universitätsklinik Köln  Klinik für Allgemein-, Viszeral-, Tumor- und Transplantationschirurgie | University Hospital Köln  Department of General, Visceral, Tumor and Transplant Surgery | Prof. Dr. Christiane Bruns |
| Universitätsklinikum Leipzig  Klinik und Poliklinik für Viszeral-, Transplantations-, Thorax- und Gefäßchirurgie | University Hospital Leipzig  Clinic and Polyclinic for Visceral, Transplant, Thoracic and Vascular Surgery | Prof. Dr. med. Ines Gockel |
| Robert-Bosch-Krankenhaus Stuttgart  Abteilung für Allgemein- und Viszeralchirurgie | Robert-Bosch-Krankenhaus Stuttgart  Department of General and Visceral Surgery | PD Dr. med. Philipp Renner |
| Universitätsklinikum Ulm  Klinik für Allgemein- und Viszeralchirurgie | University Hospital Ulm  Department of General and Visceral Surgery | Prof. Dr. med. Andre Mihaljevic |
