# Supplementary material for: Overall morbidity after total minimally invasive keyhole oesophagectomy versus hybrid oesophagectomy (the MICkey trial): study protocol for a multicentre randomized controlled trial
Source: Trials. 2023 Mar 10;24:175. doi: 10.1186/s13063-023-07134-1 (PMC9999550; doi:10.1186/s13063-023-07134-1)
Supplement: Supplementary file 2 — Additional file 2: Supplement 2. Common postoperative complications after esophagectomy and their respective definitions adapted according to the Esophagectomy Complications Consensus Group (ECCG). Grading according to Dindo-Clavien with clarifications for specific complications by the Japan Clinical Oncology Group. [file 13063_2023_7134_MOESM2_ESM.docx]

**Supplement 2**

Common postoperative complications after esophagectomy and their respective definitions adapted according to the Esophagectomy Complications Consensus Group (ECCG). Grading according to Dindo-Clavien with clarifications for specific complications by the Japan Clinical Oncology Group.

| **Complication**  (according to ECCG) | **Definition**  (according to ECCG) | **Complication-specific grading**  (according to ECCG) | **Grading according to Dindo-Clavien Classification**  (clarifications according to Japan Clinical Oncology Group) |
| --- | --- | --- | --- |
| **Pulmonary** |  |  |  |
| **Pneumonia** | Definition according to American Thoracic Society and Infectious Diseases Society of America. Am J Respir Crit Care Med. 2005 Feb 15;171(4):388-416. | **Hospital acquired pneumonia** is defined as pneumonia that occurs 48 hours or more after admission, which was not incubating at the time of admission.  **Ventilator-associated pneumonia** refers to pneumonia that arises more than 48–72 hours after endotracheal intubation  **Healthcare-associated pneumonia** includes any patient who was hospitalized in an acute care hospital for two or more days within 90 days of the infection | **Grade I:** Clinical observation or diagnostic evaluation only; intervention not indicated except for nebulizers, expectorants, or lung physiotherapy (e.g., postural drainage)  **Grade II:** Medical management indicated (e.g., antibiotics)  **Grade IIIa**: Bronchoscopic aspiration, tracheal puncture  **Grade IIIb:** Tracheostomy under general anesthesia/seda- tion or mechani- cal ventilation  **Grade IVa:** Mechanical ventilation indicated  **Grade IVb:** Sepsis or multiple organ failure  **Grade V**: Death |
| **Sars-CoV-2 infection** | Sars-CoV-2 infection proven by PCR testing with a ct-value <34 |  | I / II / IIIa / IIIb / IVa / IVb / V |
| **Pleural effusion** | Pleural effusion requiring additional drainage procedure | Although the ECCG requires Pleural effusions only to be documented when requiring additional drainage procedures, for the MICkey trial ALL pleural effusion diagnosed should be recorded and graded according to the Dindo-Clavien classification, i.e. also including grade I and II. | **Grade I:** Clinical observation or diagnostic evaluation only; intervention not indicated (drainage only through existing drainage tube)  **Grade II:** Medical management indicated (e.g., diuretics)  **Grade IIIa**: Image-guided drain placement/thoracentesis including drain replacement indicated  **Grade IIIb:** Intervention under general anesthesia indicated  **Grade IVa:** Mechanical ventilation indicated  **Grade IVb:** Multiple organ failure  **Grade V**: Death |
| **Pneumothorax** | Pneumothorax requiring treatment |  | I / II / IIIa / IIIb / IVa / IVb / V |
| **Atelectasis mucous plugging** | Atelectasis mucous plugging requiring bronchoscopy | Although the ECCG requires atelectasis mucous plugging only to be documented when requiring bronchoscopy, for the MICkey trial ALL atelectasis diagnosed should be recorded and graded according to the Dindo-Clavien classification, i.e. also including grade I and II. | **Grade I:** Clinical observation or diagnostic evaluation only; intervention not indicated, except for nebulizers, expectorants, or lung physiotherapy (e.g., postural drainage)  **Grade II:** Medical management indicated (e.g., antibiotics)  **Grade IIIa**: Bronchoscopic aspiration or surgical intervention indicated (e.g., tracheal puncture) without general anesthesia  **Grade IIIb:** Intervention under general anesthesia indicated (including tracheostomy under sedation)  **Grade IVa:** Mechanical ventilation indicated  **Grade IVb:** Sepsis or multiple organ failure  **Grade V**: Death |
| **Respiratory failure** | Respiratory failure requiring reintubation |  | IVa / IVb / V |
| **Acute respiratory distress syndrome** | Acute respiratory distress syndrome  Berlin Definition: ARDS Definition Task Force, Ranieri VM, et al. JAMA. 2012 Jun 20;307(23):2526-33.) | 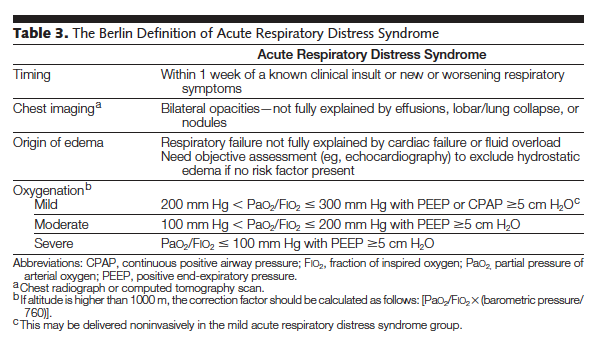 | I / II / IIIa / IIIb / IVa / IVb / V |
| **Acute aspiration** | Acute aspiration |  | I / II / IIIa / IIIb / IVa / IVb / V |
| **Tracheobronchial injury** | Tracheobronchial injury |  | I / II / IIIa / IIIb / IVa / IVb / V |
| **Air leak** | Chest tube maintenance for air leak for >10 d postoperatively |  | I / II / IIIa / IIIb / IVa / IVb / V |
| **Cardiac** |  |  |  |
| **Cardiac arrest requiring CPR** |  |  | IVa / IVb / V |
| **Myocardial infarction** | Myocardial infarction  Definition: World Health Organization: Mendis S, et al. Writing group on behalf of the participating experts of the WHO consultation for revision of WHO definition of myocardial infarction. World Health Organization definition of myocardial infarction: 2008-09 revision. Int J Epidemiol. 2011 Feb;40(1):139-46. | (i) Detection of rise and/or fall of cardiac biomarkers (preferably troponin) with at least one value above the 99th percentile of the upper reference limit together with evidence of myocardial ischaemia with at least one of the following:  (a) symptoms of ischaemia (include various combinations of chest, upper extremity, jaw or epigastric discomfort with exertion or at rest; the discomfort usually lasts 420 min, often is diffuse, not localized, not positional, not affected by movement of the region and it may be accompanied by dyspnoea, diaphoresis, nausea or syncope);  (b) ECG changes indicative of new ischaemia [new ST-T changes or new left bundle branch block (LBBB)–Minnesota codes: ST-depression 4.1; 4.2; ST-elevation 9.2; LBBB 7.1]  (c) development of pathological Q waves in the ECG (Minnesota codes: 1.1.1 through 1.2.5 plus 1.2.7),17 including: (1) no unequivocal pathological Q waves in the first ECG or in event set of ECG(s) followed by a record with a pathological Q wave or (2) any Q wave in leads V2–V3 5 0.02 s or QS complex in leads V2 and V3 or Q wave 50.03 s and 50.01 mV deep or QS complex in leads I, II, aVL, aVF or (3) V4–V6 in any two leads of a contiguous lead grouping (I, aVL, V6:V4–V6: II, III, aVF).  (d) imaging evidence of new loss of viable myocardium or new regional wall motion abnormality.  or  (ii) Sudden unexpected cardiac death, involving cardiac arrest, often with symptoms suggestive of myocardial ischaemia (ischaemic symptoms include various combinations of chest, upper extremity, jaw or epigastric discomfort with exertion or at rest; the discomfort usually lasts <20 min, often is diffuse, not localized, not positional, not affected by movement of the region, and it may be accompanied by dyspnoea, diaphoresis, nausea or syncope.) and accompanied by  (a) presumably new ST elevation or new LBBB (Minnesota codes: ST-depression 4.1; 4.2; ST-elevation 9.2; LBBB 7.1) and/or  (b) evidence of fresh thrombus by coronary angiography and/or at autopsy, but death occurring before blood samples could be obtained or at a time before the appearance of cardiac biomarkers in the blood, and there is no evidence of a non-coronary cause of death.  or  (iii) Autopsy findings of an acute MI | **Grade I:** Clinical observation or diagnostic evaluation only; intervention not indicated  **Grade II:** Medical manage- ment indicated (e.g., anticoagu lant therapy)  **Grade IIIa**: Cardiac catheterization indicated  **Grade IIIb:** Intervention under general anes- thesia indicated (coronary artery bypass)  **Grade IVa:** Heart failure associated with low cardiac output syndrome; IC/ ICU management indicated  **Grade IVb:** Heart failure associ- ated with low cardiac output syndrome and renal failure; IC/ ICU management indicated  **Grade V**: Death |
| **Dysrhythmia atrial** | Dysrhythmia atrial requiring treatment |  | **Grade I:** Clinical observation or diagnostic evaluation only; intervention not indicated  **Grade II:** Medical manage- ment indicated (e.g., antiarrhyth- mic drugs)  **Grade IIIa**: Medical intervention under local anesthesia indicated (e.g., catheter ablation, synchronized cardioversion)  **Grade IIIb:** -  **Grade IVa:** Heart failure associated with low cardiac output syndrome; IC/ ICU management indicated  **Grade IVb:** Heart failure associ-ated with low cardiac output syndrome and renal failure; IC/ ICU management indicated  **Grade V**: Death |
| **Dysrhythmia ventricular** | Dysrhythmia ventricular requiring treatment |  | **Grade I:** Clinical observation or diagnostic evaluation only; intervention not indicated  **Grade II:** Medical manage- ment indicated (e.g., antiarrhyth- mic drugs)  **Grade IIIa**: Medical interven- tion under local anesthesia indicated (e.g., catheter ablation, external defibrillator, pacemaker implanta- tion)  **Grade IIIb:** -  **Grade IVa:** Heart failure associated with low cardiac output syndrome; IC/ ICU management indicated  **Grade IVb:** Heart failure associ-ated with low cardiac output syndrome and renal failure; IC/ ICU management indicated  **Grade V**: Death |
| **Congestive heart failure** | Congestive heart failure requiring treatment |  | I / II / IIIa / IIIb / IVa / IVb / V |
| **Pericarditis** | Pericarditis requiring treatment |  | I / II / IIIa / IIIb / IVa / IVb / V |
| **Gastrointestinal** |  |  |  |
| **Anastomotic Leak** | Full thickness GI defect involving esophagus, anastomosis, staple line, or conduit irrespective of presentation or method of identification | Type I: Local defect requiring no change in therapy or treated medically or with dietary modification  Type II: Localized defect requiring interventional but not surgical therapy, for example, interventional radiology drain, stent or bedside opening, and packing of incision  Type III: Localized defect requiring surgical therapy | **Grade I:** Only small fistula observed on oral contrast study or drainage imaging (drainage only through existing drainage tube)  **Grade II:** Medical management (e.g., antibiotics) or enteral/ intravenous nutrition (Including TPN) indicated  **Grade IIIa**: Image-guided drain placement/para- centesis including wound opening or drain replacement indicated  **Grade IIIb:** Intervention under general anes- thesia indicated (e.g., suture, reanastomosis, bypass, drainage, colostomy)  **Grade IVa:** At least one organ failure (e.g., pul- monary disorders requiring mechanical ventilation or nephropathy indi- cating dialysis)  **Grade IVb:** Sepsis or multiple organ failure  **Grade V**: Death |
| **Conduit Necrosis/Failure** | Conduit Necrosis/Failure | Type I: Conduit necrosis focal. Identified endoscopically. Treatment—Additional monitoring or non-surgical therapy  Type II: Conduit necrosis focal. Identified endoscopically and not associated with free anastomotic or conduit leak. Treatment—Surgical therapy not involving esophageal diversion  Type III: Conduit necrosis extensive. Treatment—Treated with conduit resection with diversion | **Grade I:** Observation of a small fistula with oral contrast study or drainage imaging (drainage only through existing drain- age tube)  **Grade II:** Medical manage- ment (e.g., antibiotics), enteral/ intravenous nutrition indicated  **Grade IIIa**: Radiological interven- tion/endoscopic/ elective surgical intervention without general anesthesia indicated, including drain replacement  **Grade IIIb:** Intervention under general anesthesia indicated  **Grade IVa:** At least one organ failure (e.g., pul- monary disorders requiring mechanical ventilation or nephropathy indicating dialysis)  **Grade IVb:** Sepsis or multiple organ failure  **Grade V**: Death |
| **Ileus (paralytic)** | Ileus defined as small bowel dysfunction preventing or delaying enteral feeding |  | **Grade I:** Clinical observation or diagnostic evaluation only; medical management not indicated except for laxatives and intravenous nutrition  **Grade II:** Medical management beyond laxatives, NG tube placement, or intravenous nutrition management indicated  **Grade IIIa**: Nasoenteric tube placement  **Grade IIIb:** Treatment for ileus under general anesthesia (with or without intestinal resection)  **Grade IVa:** Extensive intestinal necrosis, at least one organ failure (e.g., pulmonary disorders requiring mechanical ventilation or nephropathy indicating dialysis)  **Grade IVb:** Sepsis or multiple organ failure  **Grade V**: Death |
| **Small bowel obstruction** | Small bowel obstruction |  | **Grade I:** Clinical observation or diagnostic evaluation only; medical management not indicated except for laxatives and intravenous nutrition  **Grade II:** Medical management beyond laxatives, NG tube placement, or intravenous nutrition management indicated  **Grade IIIa**: Nasoenteric tube placement  **Grade IIIb:** Treatment under general anesthesia (with or without intestinal resection)  **Grade IVa:** Extensive intestinal necrosis, at least one organ failure (e.g., pulmonary disorders requiring mechanical ventilation or nephropathy indicating dialysis)  **Grade IVb:** Sepsis or multiple organ failure  **Grade V**: Death |
| **Feeding J-tube complication** | Feeding J-tube complication |  | I / II / IIIa / IIIb / IVa / IVb / V |
| **Pyloromyotomy/pyloroplasty complication** | Pyloromyotomy/pyloroplasty complication |  | I / II / IIIa / IIIb / IVa / IVb / V |
| **Clostridium difficile Infection** | Clostridium difficile Infection |  | I / II / IIIa / IIIb / IVa / IVb / V |
| **Gastrointestinal bleeding** | Gastrointestinal bleeding requiring intervention or transfusion |  | **Grade I:** Controllable with compression only  **Grade II:** Blood transfusion or medical man- agement indicated  **Grade IIIa**: Surgical hemostasis under local anesthesia or endoscopic and radiological intervention hemostasis indicated  **Grade IIIb:** Intervention under general anes- thesia indicated (hemostasis)  **Grade IVa:** Single organ failure; stepdown ICU/ICU care indicated  **Grade IVb:** Multiple organ failure; IC/ICU management indicated  **Grade V**: Death |
| **Delayed conduit emptying** | Delayed conduit emptying requiring intervention or delaying discharge or requiring maintenance of NG drainage >7 d postoperatively |  | **Grade I:** Clinical observation or diagnostic evaluation only; intervention not indicated  **Grade II:** Medical management (e.g., peri- stalsis stimulat- ing drugs), NG tube placement, enteral/intravenous nutrition indicated  **Grade IIIa**: -  **Grade IIIb:** Intervention under general anesthesia indicated  **Grade IVa: -**  **Grade IVb: -**  **Grade V**: Death |
| **Pancreatitis** | Pancreatitis |  | I / II / IIIa / IIIb / IVa / IVb / V |
| **Liver dysfunction** | Liver dysfunction |  | I / II / IIIa / IIIb / IVa / IVb / V |
| **Urologic** |  |  |  |
| **Acute renal insufficiency** | Acute renal insufficiency (defined as doubling of baseline creatinine) |  | I / II / IIIa / IIIb / IVa / IVb / V |
| **Acute renal failure requiring dialysis** | Acute renal failure requiring dialysis |  | IVa / IVb / V |
| **Urinary tract infection** |  |  | I / II / IIIa / IIIb / IVa / IVb / V |
| **Urinary retention** | Urinary retention requiring reinsertion of urinary catheter, delaying discharge, or discharge with urinary catheter |  | **Grade I:** Intermittent catheteriza- tion or Foley catheter placement indicated  **Grade II:** Medical management indicated (e.g., cholinergics)  **Grade IIIa**: Intervention under local or lumbar anesthesia indicated (e.g., endoscopic treatment, urethral dilatation)  **Grade IIIb:** Intervention under general anes- thesia indicated (e.g., fistula closure)  **Grade IVa:** Acute renal failure, hemodialysis  **Grade IVb:** Sepsis or multiple organ failure  **Grade V**: Death |
| **Thrombembolic** |  |  |  |
| **Deep venous thrombosis** |  |  | **Grade I:** Clinical observation or diagnostic evaluation only; intervention not indicated  **Grade II:** Medical management indicated (e.g., anticoagulants)  **Grade IIIa**: Invasive treatment indicated (e.g., thrombus ablation via catheter, IVC filter)  **Grade IIIb:** Intervention under general anesthesia indicated  **Grade IVa:** Single organ failure  **Grade IVb:** Multiple organ failure  **Grade V**: Death |
| **Pulmonary embolus** |  |  | **Grade I:** Clinical observation or diagnostic evaluation only; intervention not indicated  **Grade II:** Medical management indicated (e.g., anticoagulants)  **Grade IIIa**: Invasive treatment indicated (e.g., thrombus ablation via catheter, IVC filter)  **Grade IIIb:** Intervention under general anesthesia indicated ((pulmonary artery thrombectomy)  **Grade IVa:** Single organ failure  **Grade IVb:** Multiple organ failure  **Grade V**: Death |
| **Stroke (cardiovascular accident)** |  |  | **Grade I:** Clinical observation or diagnostic evaluation only; intervention not indicated  **Grade II:** Medical management indicated (e.g., anticoagulants)  **Grade IIIa**: Radiological interven- tion without general anesthesia (e.g., intracerebrovascular treatment)  **Grade IIIb:** Intervention under general anes- thesia indicated (e.g., drainage, surgical clipping, cerebrovascular bypass, carotid endarterectomy)  **Grade IVa: I**C/ICU management indicated  **Grade IVb:** IC/ICU management indicated; associated with respiratory failure  **Grade V**: Death |
| **Peripheral thrombophlebitis** |  |  | I / II / IIIa / IIIb / IVa / IVb / V |
| **Neurologic / psychiatric** |  |  |  |
| **Recurrent nerve injury/**  **Vocal Cord Injury/**  **Vocal Cord Palsy** | Vocal cord dysfunction post-resection. Confirmation and assessment should be by direct examination | Type I: Transient injury requiring no therapy. Dietary modification allowed  Type II: Injury requiring elective surgical procedure, for example, thyroplasty or medialization procedure  Type III: Injury requiring acute surgical intervention (due to aspiration or respiratory issues), for example, thyroplasty or medialization procedure  Severity Level: (A) Unilateral vs. (B) Bilateral | **Grade I**: Clinical observation or diagnostic evaluation only; intervention not indicatede  **Grade II:** Aspiration; medi- cal management indicated (e.g., antibiotics)  **Grade IIIa**: Severe aspiration; food intake almost impossible; medical intervention under local anesthesia indicated (e.g., vocal cord injection, tracheal puncture)  **Grade IIIb:** Intervention under general anes- thesia indicated (including tra- cheostomy under sedation)  **Grade IVa:** Mechanical ventila- tion indicated  **Grade IVb:** Sepsis or multiple organ failure  **Grade V**: Death |
| **Other neurologic injury** |  |  | I / II / IIIa / IIIb / IVa / IVb / V |
| **Acute delirium** | Definition: Diagnostic and Statistical Manual of Mental Disorders, 5th ed | All of the following must be fulfilled:  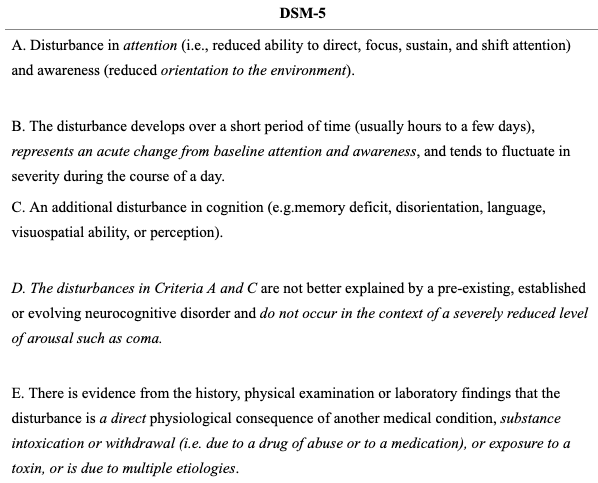 | I / II / IIIa / IIIb / IVa / IVb / V |
| **Delirium tremens** | Definition: Diagnostic and Statistical Manual of Mental Disorders, 5th ed | **Definition of delirium** (see above)  **AND**  **Criteria for alcohol withdrawal:** Cessation of or reduction in heavy and prolonged use of alcohol. At **least 2 of 8 possible symptoms** after reduced use of alcohol:   - Autonomic hyperactivity - Hand tremor - Insomnia - Nausea or vomiting - Transient hallucinations or illusions - Psychomotor agitation - Anxiety - Generalized tonic–clonic seizures | I / II / IIIa / IIIb / IVa / IVb / V |
| **Infection** |  |  |  |
| **Wound infection** | Wound infection requiring opening wound or antibiotics |  | **Grade I**: Clinical observation or diagnostic evaluation only; intervention not indicated, except for wound opening and wound irrigation at the bedside  **Grade II:** Medical manage- ment indicated (e.g., antibiotics)  **Grade IIIa**: Medical intervention under local anesthe- sia indicated (e.g., drainage)  **Grade IIIb:** Intervention under general anesthesia indicated (e.g., drainage, resuturing)  **Grade IVa:** At least one organ failure (e.g., pul monary disorders requiring mechanical ventilation or nephropathy indicating dialysis)  **Grade IVb:** Sepsis or multiple organ failure  **Grade V**: Death |
| **Central catheter infection** | Central IV line infection requiring removal or antibiotics |  | I / II / IIIa / IIIb / IVa / IVb / V |
| **Intrathoracic/intra-abdominal abscess** | Intrathoracic/intra-abdominal abscess |  | I / II / IIIa / IIIb / IVa / IVb / V |
| **Generalized sepsis** | Definition according to SEPSIS-3. JAMA. 2016 Feb 23; 315(8): 801–810. | Sepsis is defined as life-threatening organ dysfunction caused by a dysregulated host response to infection. For clinical operationalization, organ dysfunction can be represented by an increase in the Sequential [Sepsis-related] Organ Failure Assessment (**SOFA**) **score of 2 points or more:**  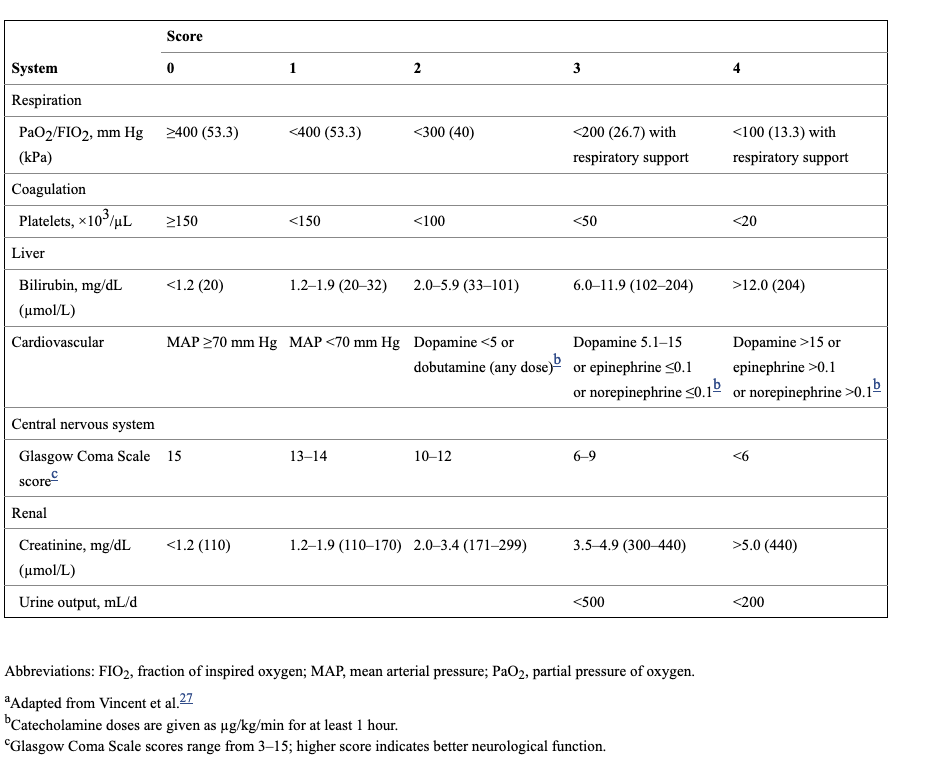  or at **least 2 of the following clinical criteria** (quickSOFA, = screening tool):   1. respiratory rate of 22/min or greater, 2. altered mentation, or 3. systolic blood pressure of 100 mm Hg or less. | IVa / IVb / V |
| **Other infection** | Other infections requiring antibiotics |  | I / II / IIIa / IIIb / IVa / IVb / V |
| **Wound / diaphgram** |  |  |  |
| **Thoracic wound dehiscence** | Thoracic wound dehiscence |  | I / II / IIIa / IIIb / IVa / IVb / V |
| **Acute abdominal wall dehiscence/hernia/ “Platzbauch”** | Acute abdominal wall dehiscence/hernia/ “Platzbauch” |  | I / II / IIIa / IIIb / IVa / IVb / V |
| Acute diaphragmatic hernia | Acute diaphragmatic hernia |  | I / II / IIIa / IIIb / IVa / IVb / V |
| **Other** |  |  |  |
| **Chyle leak** | Chyle leak | Type I: Treatment—enteric dietary modifications  Type II: Treatment—total parenteral nutrition  Type III: Treatment—interventional or surgical therapy (Does not include elective insertion of additional surgical or interventional chest drains)  Severity Level: (A) <1 liter output/day vs. (B) >1 liter output/day | **Grade I**: Observation of chylous drainage fluid or thoracentesis fluid only (drainage only through existing drainage tube)  **Grade II:** Fat-restricted diet, intravenous nutri- tion indicated  **Grade IIIa**: Image-guided drain placement/para- centesis including drain replacement indicated  **Grade IIIb:** Intervention under general anesthesia indicated (e.g., thoracic duct ligation)  **Grade IVa:** -  **Grade IVb:** -  **Grade V**: Death |
| **Reoperation for reasons other than bleeding, anastomotic leak, or conduit necrosis** | Reoperation for reasons other than bleeding, anastomotic leak, or conduit necrosis |  | I / II / IIIa / IIIb / IVa / IVb / V |
| **Other complication** | Free text |  | I / II / IIIa / IIIb / IVa / IVb / V |
